# Prognostic value of γ‐glutamyltransferase‐to‐albumin ratio in patients with pancreatic ductal adenocarcinoma following radical surgery

**DOI:** 10.1002/cam4.1957

**Published:** 2019-01-10

**Authors:** Shuo Li, Huaxiang Xu, Chuntao Wu, Wenquan Wang, Wei Jin, Heli Gao, Hao Li, Shirong Zhang, Jinzhi Xu, Wuhu Zhang, Shuaishuai Xu, Tianjiao Li, Quanxing Ni, Xianjun Yu, Liang Liu

**Affiliations:** ^1^ Department of Pancreatic Surgery Fudan University Shanghai Cancer Center Shanghai China; ^2^ Department of Oncology Shanghai Medical College Fudan University Shanghai China; ^3^ Shanghai Pancreatic Cancer Institute Shanghai China; ^4^ Pancreatic Cancer Institute Fudan University Shanghai China

**Keywords:** γ‐glutamyltransferase‐to‐albumin ratio, overall survival, pancreatic ductal adenocarcinoma, prognosis, recurrence‐free survival

## Abstract

Pancreatic ductal adenocarcinoma (PDAC) is a devastating malignancy with poor prognosis. Many preoperative biomarkers can predict postoperative survival of PDAC patients. In this study, we created a novel ratio index based on preoperative liver function test, γ‐glutamyltransferase‐to‐albumin ratio (GAR), and evaluated its prognostic value in predicting clinical outcomes of PDAC patients following radical surgery. We retrospectively enrolled 833 PDAC patients who had underwent radical surgery at our institution between January 2010 and January 2017. Patients were divided into two groups according to the cut‐off value of GAR. Univariate and multivariate survival analysis between the groups were evaluated. TNM stage, GAR, preoperative serum carbohydrate antigen 19‐9 (CA19‐9) and tumor differentiation were combined to generate a more accurate prognostic model. The optimal cut‐off value of GAR was 0.65. Significant correlations were found between GAR and tumor location, tumor size, vascular invasion, obstructive jaundice, biliary drainage and parameters of liver function test. Univariate and multivariate analysis showed that high level of GAR independently predicted poorer postoperative overall survival (OS, *P * < 0.001) and recurrence‐free survival (RFS, *P * < 0.001). Subgroup analysis demonstrated that GAR was predictive of survival in patients without biliary obstruction or severely impaired liver function. In addition, integration of GAR, preoperative serum CA19‐9, and tumor differentiation into TNM staging system could better stratify the prognosis for PDAC patients compared with TNM stage alone. Our study demonstrates that preoperative GAR is an independent prognostic factor for prediction of surgical outcomes in PDAC patients. Combination of TNM stage, GAR, preoperative serum CA19‐9, and tumor differentiation can enhance the prognostic accuracy.

## 1 INTRODUCTION

Pancreatic cancer is one of the most lethal malignancies worldwide, with a 5‐year survival rate of 8% with all stages combined.^1^ In 2018, there will be approximately 55 440 new cases of pancreatic cancer and 44 330 pancreatic cancer‐related deaths in the United States, and pancreatic cancer is estimated to rank fourth among all causes of cancer death.^1^ In China, the incidence rate for pancreatic cancer has been increasing sharply in the past decade, and it is now the ninth leading cause of cancer‐related mortalities.^2^ Radical resection is the only option for a curative treatment. However, even for patients underwent curative surgery, the 5‐year survival rate is only around 25%.^3^

Lack of detective biomarkers for early‐stage pancreatic cancer and high incidence of local recurrence and distant metastasis are two main reasons for the poor outcome of this disease. Currently, the prediction of survival and tumor recurrence for resectable pancreatic cancer patients mainly relies on histopathological features of tumor specimen, such as tumor size, lymph node metastasis, tumor differentiation, and resection margin.^4^, ^5^, ^6^ However, these predictors are only available for evaluation postoperatively, which are costly and time consuming and make it difficult for survival prediction before surgery. Besides, patients with the same TNM stage usually exhibit different clinical outcomes, therefore causing confusion among clinicians when making further treatment strategies. Serum carbohydrate antigen 19‐9 (CA19‐9) is a well‐established predictive tumor biomarker in pancreatic cancer. An elevated level of preoperative serum CA19‐9 is associated with poor prognosis.^7^ However, around 5% to 14% of the population is CA19‐9 nonsecretory phenotype, which limits the clinical use of CA19‐9 alone in certain group of patients.^8^ Liver function test is a basic routine examination before surgery. Some of its components, including alanine aminotransferase (ALT), albumin (ALB), and alkaline phosphatase (ALP), have been shown to have prognostic values for postoperative pancreatic cancer patients.^9^, ^10^ Therefore, to provide better prognostic indicators in patients with resectable pancreatic cancer, it is of interest to further dig into parameters of liver function test and identify potential preoperative biomarkers that can predict postoperative survival.

γ‐glutamyltransferase (GGT) is an important enzyme conventionally assessed in liver function test. It is widely distributed on the luminal surface of most secretory epithelial cells, especially hepatocytes and cholangiocytes.^11^ GGT plays a key role in the metabolism of glutathione (GSH), the major intracorporal antioxidant, and maintain its adequate level, therefore protecting cells from oxidative stress produced under physiological and pathological conditions.^11^ Elevated GGT is commonly seen in hepatic and biliary diseases.^12^, ^13^ It is also implicated in cardiovascular disease, type 2 diabetes mellitus, and hypertension.^14^ High level of GGT is an early marker of oxidative stress and a predictor of increased cancer risk.^15^ More importantly, increasing evidence has suggested that high level of serum GGT is associated with poor prognosis in different types of cancers, such as pancreatic cancer, cervical cancer, renal cell carcinoma, and prostate cancer.^16^, ^17^, ^18^, ^19^ ALB is synthesized in the polysomes of hepatocytes and reflects liver reserve ability. It is crucial in multiple physiological processes, including maintenance of the colloid osmotic pressure, drug delivery, scavenging of oxygen free radical, and participation in intracellular signaling pathways.^20^ Hypoalbuminemia usually occurs when liver function is impaired.^21^ It also has diagnostic and prognostic values in various types of cancers, including hepatocellular carcinoma, pancreatic cancer, and breast cancer.^22^, ^23^, ^24^ Specifically, some ALB‐based ratio index have been identified as independent prognostic factors for pancreatic cancer patients, including C‐reactive protein/albumin (CRP/ALB) ratio and platelet‐to‐albumin ratio (PAR).^25^, ^26^

Therefore, we reasonably combined the above two parameters and created a novel serological marker, γ‐glutamyltransferase‐to‐albumin ratio (GAR), based on preoperative liver function test. It is easily accessible, time saving and can be obtained from all resectable patients before surgery. The purpose of this study is to explore the predictive value of GAR on postoperative survival in patients with resectable pancreatic ductal adenocarcinoma (PDAC) and further assess whether combination of GAR with other prognostic factors can improve prognostic accuracy.

## 2 PATIENTS AND METHODS

### 2.1 Patients selection and data collection

A total of 833 eligible patients who underwent radical operation for PDAC from January 2010 to January 2017 at Department of Pancreatic Surgery, Fudan University, Shanghai Cancer Center were collected. The inclusion and exclusion criteria were as follows: (a) pathologically proven PDAC; (b) no preoperative antitumor treatment; (c) no history of other malignant tumors; (d) complete clinicopathologic and follow‐up data after operation; (e) negative resection margin demonstrated by pathological examination; (f) no evidence of distant metastasis at the time of surgery; (g) no perioperative death caused by severe surgical complications. The following clinicopathologic variables were collected in this study: gender, age, tumor location, tumor size, lymph node metastasis, TNM stage, tumor differentiation, vascular invasion, obstructive jaundice, biliary drainage, and laboratory tests including blood routine, CA19‐9, ALP, ALT, aspartate aminotransferase (AST), GGT, ALB, and glucose. Blood samples for laboratory tests were collected and analyzed within 7 days before operation. The clinical staging was determined by TNM staging system of the American Joint Commission on Cancer (AJCC) 8th edition via clinical evaluation and postoperative pathological examination. GAR was calculated as the serum GGT level divided by the serum ALB level. This study was approved by the Human Research Ethics Committee of Fudan University Shanghai Cancer Center and was in accordance with the tenets of the World Medical Association Declaration of Helsinki. Informed consent was obtained from all patients according to the committee's regulations.

### 2.2 Follow‐up

All patients were regularly followed up after surgery. Physical and laboratory examinations were carried out for each patient every 3 months. Enhanced abdominal computed tomography scan (CT) or magnetic resonance imaging (MRI) were routinely performed every 6 months. If local recurrence or distant metastasis was suspected, image examinations including CT, MRI, bone scans, and positron emission tomography‐computed tomography (PET‐CT) were selectively conducted immediately. Overall survival (OS) was defined as the interval between the date of surgery and death or the last follow‐up visit. Recurrence‐free survival (RFS) was defined as the interval between the date of surgery and tumor recurrence or the last follow‐up visit. The last follow‐up time was October 2017.

### 2.3 Statistical analysis

All statistical analyses were performed using SPSS 21.0 (Chicago, IL, USA). The optimal cut‐off value for GAR was determined by receiver operating characteristic curve (ROC) analysis. The correlations between GAR and clinicopathologic variables were analyzed by Pearson Chi‐squared test, Fisher's exact test or Mann‐Whitney *U* test as appropriate. The Cox proportional hazard regression model was used for univariate and multivariate analyses. Survival curves were plotted according to the Kaplan‐Meier method and differences between subgroups were compared using the log‐rank test. The concordance index (C‐index) and Akaike information criterion (AIC) were calculated by Stata/SE 11.0 (Texas, USA). *P* values <0.05 (two‐sided) were considered statistically significant.

## 3 RESULTS

### 3.1 Clinicopathologic characteristics

Detailed clinicopathologic characteristics of all enrolled patients are summarized in Table 1. Of the entire study population, 465 were males and 368 were females. The median age was 61 years (range 33‐84 years). In total, 466 patients had tumors located at the pancreatic head, whereas the remaining had tumors located at the body or tail of the pancreas. The size of tumor was not more than 4 cm in 605 patients and lymph node metastasis was present in 408 patients. According to TNM staging system of the AJCC 8th edition, the number of patients classified into I, II, and III stages were 322, 407, and 104, respectively. A normal level of preoperative serum CA19‐9 was observed in 196 patients. A total of 226 patients had preoperative obstructive jaundice, and 148 of them received biliary drainage before surgery.

**Table 1:** Correlations between GAR and clinicopathologic features of PDAC patients

Variables	Cases	GAR ≤ 0.65	GAR > 0.65	*P*
Total number	833	338	495	
Gender				
Female	368	155	203	0.576
Male	465	183	292	
Age (y)				
≤61	424	163	261	0.202
>61	409	175	234	
Tumor location				
Head	466	92	374	<0.001
Body or tail	367	246	121	
Tumor size (cm)				
≤4	605	220	385	<0.001
>4	228	118	110	
Lymph node status				
Negative	425	176	249	0.616
Positive	408	162	246	
TNM stage				
I	322	125	197	0.445
II	407	174	233	
III	104	39	65	
Tumor differentiation				
Well to moderate	534	221	313	0.525
Poor	299	117	182	
Vascular invasion				
No	633	275	358	0.003
Yes	200	63	137	
CA19‐9 (U/mL)				
≤37	196	87	109	0.214
>37	637	251	386	
Obstructive jaundice				
No	607	337	270	<0.001
Yes	226	1	225	
Biliary drainage				
No	685	338	347	<0.001
Yes	148	0	148	
ALP (U/L)				
≤100	466	307	159	<0.001
>100	367	31	336	
ALT (U/L)				
≤40	529	324	205	<0.001
>40	304	14	290	
AST (U/L)				
≤35	589	333	256	<0.001
>35	244	5	239	
GGT (U/L)				
≤40	451	338	113	<0.001
>40	382	0	382	
ALB (g/L)				
≤35	186	42	144	<0.001
>35	647	296	351	
Glucose (mmol/L)				
≤6.1	414	177	237	0.203
>6.1	419	161	258	

ALB, albumin; ALP, alkaline phosphatase; ALT, alanine aminotransferase; AST, aspartate aminotransferase; CA19‐9, carbohydrate antigen 19‐9; GAR, γ‐glutamyltransferase‐to‐albumin ratio; GGT, γ‐glutamyltransferase; PDAC, pancreatic ductal adenocarcinoma.

All patients were followed up until October 2017. At the time of last follow‐up, 505 patients were confirmed died. The median OS time was 20.8 months, and the OS rates at 1, 2, and 3 years were 80.7%, 42.5%, and 26.4%, respectively. The median RFS time was 10.7 months, and the RFS rates at 1, 2, and 3 years were 46.3%, 24.9%, and 20.5%, respectively.

### 3.2 The relationship between GAR and clinicopathologic factors in PDAC patients

The optimal cut‐off value for GAR was calculated to be 0.65 for survival prediction by ROC analysis, ranging from 0.12 to 69.92. Patients were stratified into two groups according to the value of GAR (low level of GAR, ≤0.65, n = 338 and high level of GAR, >0.65, n = 495). Analysis of the relationship between GAR and other clinicopathologic factors in PDAC patients are shown in Table 1. High level of GAR was significantly associated with pancreatic head cancer (*P *<* *0.001), large tumor size (*P *<* *0.001), presence of vascular invasion (*P *=* *0.003), presence of obstructive jaundice (*P *<* *0.001), preoperative biliary drainage (*P *<* *0.001), high level of ALP (*P *<* *0.001), high level of ALT (*P *<* *0.001), high level of AST (*P *<* *0.001), high level of GGT (*P *<* *0.001), and low level of ALB (*P *<* *0.001).

### 3.3 Independent prognostic factors for OS and RFS

**Figure 1:**
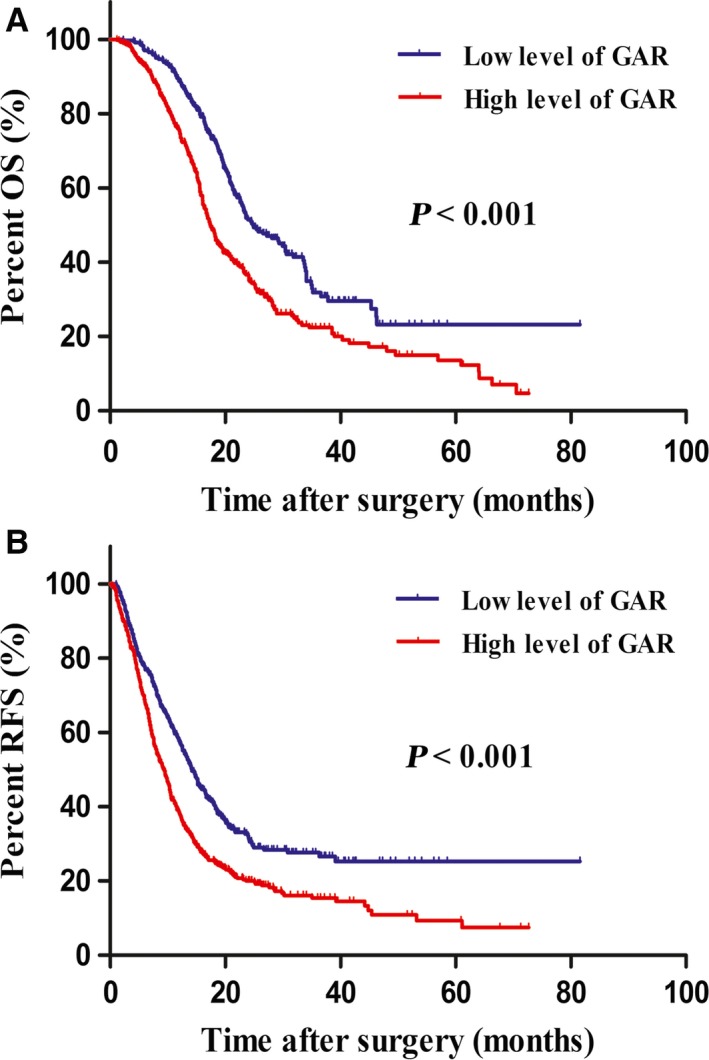
Kaplan‐Meier survival curves for overall survival (OS) and recurrence‐free survival (RFS) according to γ‐glutamyltransferase‐to‐albumin ratio (GAR) in patients with pancreatic ductal adenocarcinoma (PDAC). Patients with low level of GAR were associated with significantly better OS (A) and RFS (B) compared with patients with high level of GAR

In univariate analysis, high level of GAR was identified as adverse prognostic factors for OS (HR = 1.724, *P *<* *0.001) and RFS (HR = 1.527, *P *<* *0.001). The median OS of patients with high level of GAR was 7.4 months shorter than those with low level of GAR (17.4 months vs 24.8 months). The 1, 2, and 3‐year OS rates of patients with high level of GAR were significantly lower than patients with low level of GAR (75.2%, 36.2%, and 22.4% vs 88.7%, 51.4%, and 31.9%, respectively; *P *<* *0.001; Figure 1A). The median RFS of patients with high level of GAR was 4.8 months shorter than those with low level of GAR (9.4 months vs 14.2 months). The 1, 2, and 3‐year RFS rates of patients with high level of GAR were significantly lower than patients with low level of GAR (38.1%, 20.0%, and 15.4% vs 57.8%, 31.6%, and 27.7%, respectively; *P *<* *0.001; Figure 1B).

In addition, large tumor size (HR = 1.554, *P *<* *0.001), presence of lymph node metastasis (HR = 1.811, *P *<* *0.001) and vascular invasion (HR = 1.556, *P *<* *0.001), advanced TNM stage (HR = 1.854, *P *<* *0.001; HR = 3.465, *P *<* *0.001), poor tumor differentiation (HR = 1.464, *P *<* *0.001), presence of preoperative obstructive jaundice (HR = 1.278, *P *=* *0.013), elevated preoperative serum CA19‐9 (HR = 1.443, *P *=* *0.001), high level of ALT (HR = 1.312, *P *=* *0.003), high level of AST (HR = 1.265, *P *=* *0.015), and high level of GGT (HR = 1.487, *P *<* *0.001) were also predictive of poor OS in univariate analysis (Table 2). Similarly, the adverse prognostic factors for RFS included large tumor size (HR = 1.437, *P *<* *0.001), presence of lymph node metastasis (HR = 1.717, *P *<* *0.001) and vascular invasion (HR = 1.400, *P *<* *0.001), advanced TNM stage (HR = 1.652, *P *<* *0.001; HR = 2.697, *P *<* *0.001), poor tumor differentiation (HR = 1.405, *P *<* *0.001), presence of preoperative obstructive jaundice (HR = 1.215, *P *=* *0.027), elevated preoperative serum CA19‐9 (HR = 1.500, *P *<* *0.001), high level of ALT (HR = 1.251, *P *=* *0.006), high level of AST (HR = 1.258, *P *=* *0.008), and high level of GGT (HR = 1.393, *P *<* *0.001) (Table 3).

**Table 2:** Univariate and multivariate analysis for OS in PDAC patients

Variables	Univariate analysis			Multivariate analysis		
	HR	95% CI	*P*	HR	95% CI	*P*
Gender						
Female	1					
Male	1.161	0.927‐1.386	0.100			
Age (y)						
≤61	1					
>61	1.066	0.895‐1.269	0.474			
Tumor location						
Head	1					
Body or tail	0.859	0.719‐1.025	0.092			
Tumor size (cm)						
≤4	1					
>4	1.554	1.283‐1.882	<0.001			
Lymph node status						
Negative	1					
Positive	1.811	1.518‐2.160	<0.001			
TNM stage						
I	1			1		
II	1.854	1.526‐2.251	<0.001	1.953	1.602‐2.381	<0.001
III	3.465	2.622‐4.578	<0.001	3.395	2.531‐4.554	<0.001
Tumor differentiation						
Well to moderate	1			1		
Poor	1.464	1.226‐1.750	<0.001	1.499	1.249‐1.799	<0.001
Vascular invasion						
No	1			1		
Yes	1.556	1.278‐1.894	<0.001	1.144	0.929‐1.410	0.206
CA19‐9 (U/mL)						
≤37	1			1		
>37	1.443	1.163‐1.791	0.001	1.331	1.071‐1.655	0.010
Obstructive jaundice						
No	1			1		
Yes	1.278	1.054‐1.550	0.013	0.975	0.727‐1.308	0.867
Biliary drainage						
No	1					
Yes	1.132	0.894‐1.433	0.303			
ALP (U/L)						
≤100	1					
>100	1.183	0.992‐1.411	0.061			
ALT (U/L)						
≤40	1			1		
>40	1.312	1.096‐1.569	0.003	1.026	0.753‐1.398	0.870
AST (U/L)						
≤35	1			1		
>35	1.265	1.047‐1.528	0.015	0.851	0.618‐1.171	0.322
GGT (U/L)						
≤40	1			1		
>40	1.487	1.247‐1.772	<0.001	1.069	0.781‐1.464	0.677
ALB (g/L)						
≤35	1					
>35	0.836	0.682‐1.025	0.085			
Glucose (mmol/L)						
≤6.1	1					
>6.1	1.073	0.901‐1.278	0.429			
GAR						
≤0.65	1			1		
>0.65	1.724	1.434‐2.072	<0.001	1.882	1.434‐2.471	<0.001

ALB, albumin; ALP, alkaline phosphatase; ALT, alanine aminotransferase; AST, aspartate aminotransferase; CA19‐9, carbohydrate antigen 19‐9; CI, confidence interval; GAR, γ‐glutamyltransferase‐to‐albumin ratio; GGT, γ‐glutamyltransferase; HR, hazard ratio; OS, overall survival; PDAC, pancreatic ductal adenocarcinoma.

**Table 3:** Univariate and multivariate analysis for RFS in PDAC patients

Variables	Univariate analysis			Multivariate analysis		
	HR	95% CI	*P*	HR	95% CI	*P*
Gender						
Female	1					
Male	1.115	0.952‐1.306	0.177			
Age (y)						
≤61	1					
>61	0.942	0.805‐1.101	0.451			
Tumor location						
Head	1					
Body or tail	0.919	0.784‐1.076	0.293			
Tumor size (cm)						
≤4	1					
>4	1.437	1.209‐1.708	<0.001			
Lymph node status						
Negative	1					
Positive	1.717	1.468‐2.010	<0.001			
TNM stage						
I	1			1		
II	1.652	1.391‐1.963	<0.001	1.650	1.385‐1.965	<0.001
III	2.697	2.107‐3.453	<0.001	2.511	1.937‐3.256	<0.001
Tumor differentiation						
Well to moderate	1			1		
Poor	1.405	1.197‐1.650	<0.001	1.433	1.216‐1.688	<0.001
Vascular invasion						
No	1			1		
Yes	1.400	1.172‐1.674	<0.001	1.070	0.887‐1.292	0.480
CA19‐9 (U/mL)						
≤37	1			1		
>37	1.500	1.236‐1.822	<0.001	1.385	1.138‐1.686	0.001
Obstructive jaundice						
No	1			1		
Yes	1.215	1.022‐1.444	0.027	0.906	0.694‐1.183	0.468
Biliary drainage						
No	1					
Yes	1.107	0.902‐1.359	0.329			
ALP (U/L)						
≤100	1					
>100	1.128	0.964‐1.320	0.133			
ALT (U/L)						
≤40	1			1		
>40	1.251	1.065‐1.468	0.006	1.007	0.763‐1.329	0.961
AST (U/L)						
≤35	1			1		
>35	1.258	1.063‐1.490	0.008	0.986	0.738‐1.317	0.923
GGT (U/L)						
≤40	1			1		
>40	1.393	1.190‐1.630	<0.001	1.059	0.798‐1.406	0.691
ALB (g/L)						
≤35	1					
>35	0.860	0.715‐1.033	0.107			
Glucose (mmol/L)						
≤6.1	1					
>6.1	0.955	0.817‐1.117	0.566			
GAR						
≤0.65	1			1		
>0.65	1.527	1.297‐1.797	<0.001	1.552	1.215‐1.984	<0.001

ALB, albumin; ALP, alkaline phosphatase; ALT, alanine aminotransferase; AST, aspartate aminotransferase; CA19‐9, carbohydrate antigen 19‐9; CI, confidence interval; GAR, γ‐glutamyltransferase‐to‐albumin ratio; GGT, γ‐glutamyltransferase; HR, hazard ratio; PDAC, pancreatic ductal adenocarcinoma; RFS, recurrence‐free survival.

Multivariate analysis demonstrated that advanced TNM stage (HR = 1.953, *P *<* *0.001, and HR = 3.395, *P *<* *0.001 for OS; HR = 1.650, *P *<* *0.001, and HR = 2.511, *P *<* *0.001 for RFS), high level of GAR (HR = 1.882, *P *<* *0.001 for OS; HR = 1.552, *P *<* *0.001 for RFS), elevated preoperative serum CA19‐9 (HR = 1.331, *P *=* *0.010 for OS; HR = 1.385, *P *=* *0.001 for RFS), and poor tumor differentiation (HR = 1.499, *P *<* *0.001 for OS; HR = 1.433, *P *<* *0.001 for RFS) were independent adverse prognostic factors, for both OS and RFS prediction (Tables 2 and 3).

### 3.4 Prognostic value of GAR in different subgroups

**Figure 2:**
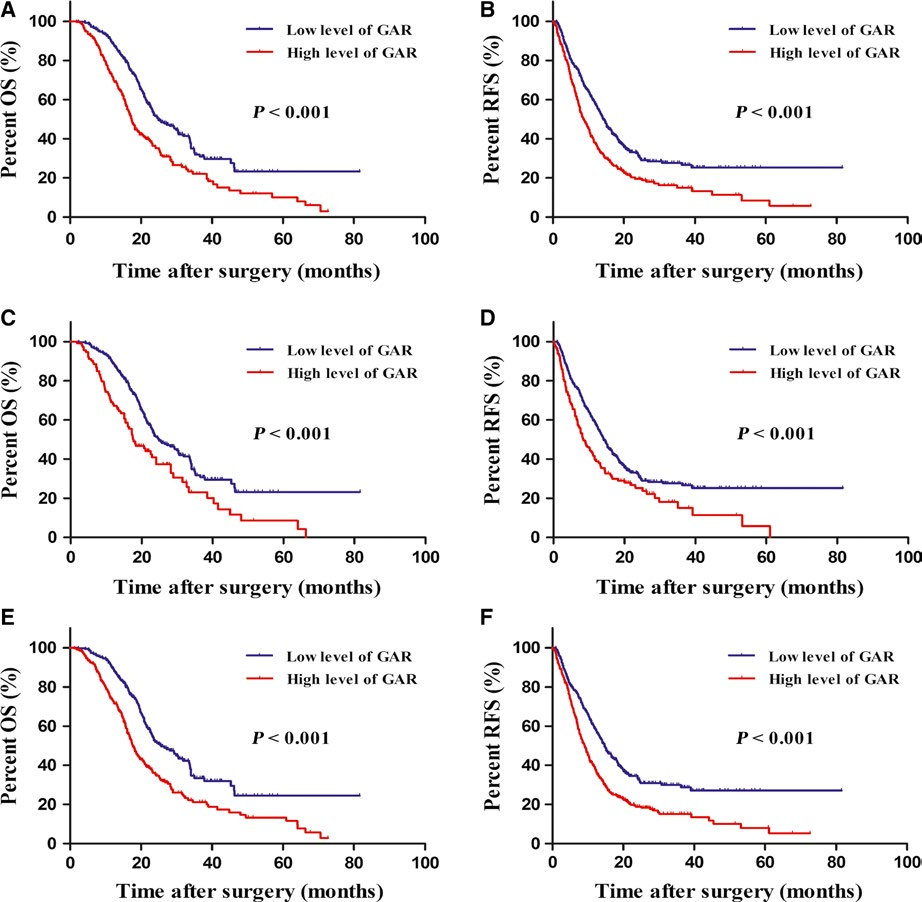
Kaplan‐Meier survival curves for overall survival (OS) and recurrence‐free survival (RFS) in patients with pancreatic ductal adenocarcinoma (PDAC) according to preoperative abnormalities of obstructive jaundice, γ‐glutamyltransferase, and albumin. Low level of γ‐glutamyltransferase‐to‐albumin ratio (GAR) was associated with significantly better OS and RFS in patients who had no abnormalities of preoperative obstructive jaundice (A and B), γ‐glutamyltransferase (C and D), or albumin (E and F)

According to whether patients had preoperative obstructive jaundice and abnormalities of GGT or ALB, we further investigated the predictive effect of GAR in each different subgroups. The results showed that high level of GAR was a significant prognostic indicator of poorer OS (24.9 months vs 17.3 months, *P *<* *0.001, Figure 2A) and RFS (14.2 months vs 8.7 months, *P *<* *0.001, Figure 2B) in patients without preoperative obstructive jaundice. Furthermore, in patients with normal level of GGT, GAR >0.65 had notable prognostic value in predicting poorer OS (24.6 months vs 17.5 months, *P *<* *0.001, Figure 2C) and RFS (14.1 months vs 8.2 months, *P *<* *0.001, Figure 2D), and this prognostic value of OS (25.4 months vs 17.6 months, *P *<* *0.001, Figure 2E) and RFS (14.7 months vs 9.1 months, *P *<* *0.001, Figure 2F) also existed in patients without ALB abnormality. However, we could not find out the association of GAR and prognosis in patients with any one abnormality of preoperative jaundice, GGT, or ALB.

**Figure 3:**
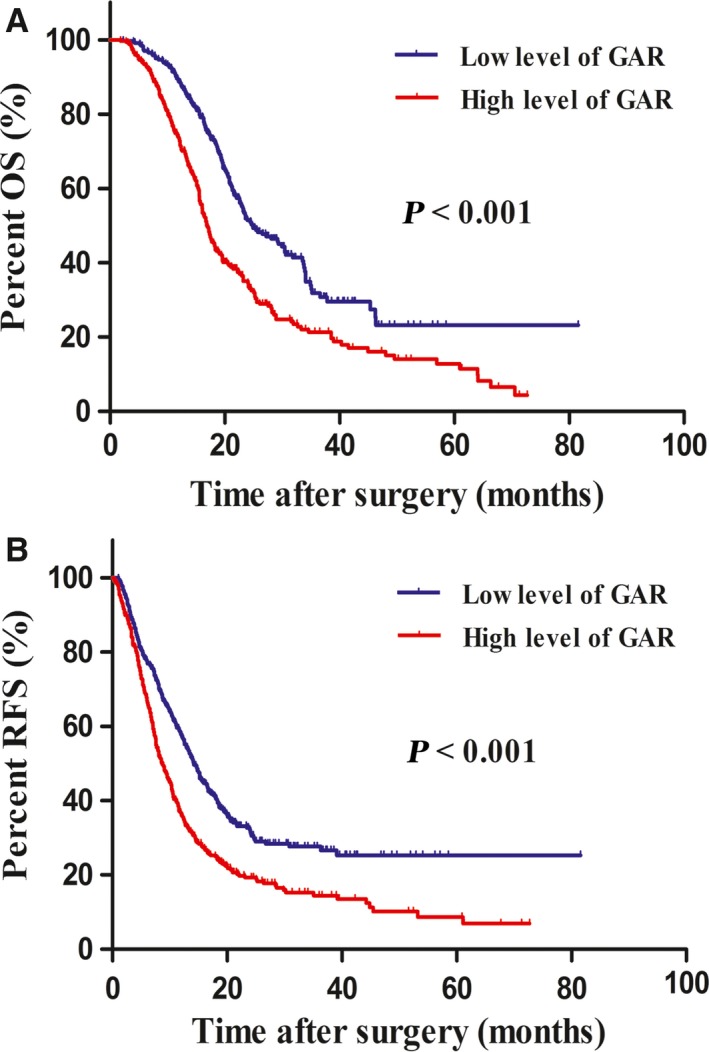
Kaplan‐Meier survival curves for overall survival (OS) and recurrence‐free survival (RFS) in patients with pancreatic ductal adenocarcinoma (PDAC) according to preoperative biliary drainage. Low level of γ‐glutamyltransferase‐to‐albumin ratio (GAR) was associated with significantly better OS and RFS in patients who did not have preoperative biliary drainage (A and B)

When stratified by preoperative biliary drainage, we found that among patients who did not have biliary drainage, those with low level of GAR had significantly longer OS (24.6 months vs 16.8 months, *P *<* *0.001, Figure 3A) and RFS (14.1 months vs 8.6 months, *P *<* *0.001, Figure 3B) than those with high level of GAR. However, we failed to confirm the prognostic value of GAR in patients who received preoperative biliary drainage. The predictive effect of GAR was therefore limited in this group of patients.

### 3.5 Combination of TNM stage, GAR, preoperative serum CA19‐9, and tumor differentiation enhances prognostic accuracy for PDAC patients

Multivariate analysis revealed that TNM stage, GAR, preoperative serum CA19‐9, and tumor differentiation were four independent prognostic factors for both OS and RFS in PDAC patients. We therefore combined these four parameters to generate a more accurate prognostic model. The C‐indices and AIC values of all parameters and their combinations are shown in Table 4. The C‐indices of TNM stage combined with GAR in OS and RFS prediction were 0.6727 and 0.6348, respectively. Corresponding AIC values were 5956 and 7591. When combining all four parameters, the C‐indices in OS and RFS prediction were 0.6923 and 0.6559, respectively. Corresponding AIC values were 5932 and 7564. Thus, combination of TNM stage, GAR, preoperative serum CA19‐9, and tumor differentiation can enhance the prognostic accuracy for OS and RFS in patients with PDAC.

**Table 4:** C‐indices and AIC values of TNM stage, GAR, preoperative serum CA19‐9, tumor differentiation, and their combinations in OS and RFS prediction

Variables	OS		RFS	
	C‐index	AIC	C‐index	AIC
TNM stage	0.6339	5997	0.6079	7617
GAR	0.5822	6042	0.5549	7657
CA19‐9	0.5322	6065	0.5389	7666
Tumor differentiation	0.5555	6060	0.5499	7667
TNM stage + GAR	0.6727	5956	0.6348	7591
TNM stage + GAR + CA19‐9+ tumor differentiation	0.6923	5932	0.6559	7564

AIC, Akaike information criterion; CA19‐9, carbohydrate antigen 19‐9; C‐index, concordance index; GAR, γ‐glutamyltransferase‐to‐albumin ratio; OS, overall survival; RFS, recurrence‐free survival.

## 4 DISCUSSION

It is now becoming clear that inflammation is a critical component in tumor initiation and progression.^27^ Oxidative stress, commonly seen in the tumor microenvironment, can activate a series of transcription factors, which lead to expression of pro‐inflammatory molecules, therefore promoting transformation of normal cells to tumor cells, tumor cell survival, proliferation, and invasion.^28^ As an essential part of the cellular defense system, GGT plays a pivotal role in maintaining sufficient level of GSH, the latter of which protects the cells from oxidative damage. GGT has been demonstrated to be elevated under pathological status of oxidative stress, and it is now regarded as a robust indicator of oxidative stress.^11^ Diergaarde et al^29^ demonstrated that a common variation in the GGT1 gene was involved in pancreatic carcinogenesis and might affect the risk of pancreatic cancer. Compared with normal pancreas and stellate cells, pancreatic tumor cells, and tumor‐associated stellate cells express higher levels of GGT.^30^ In addition, Engelken et al^16^ demonstrated that elevated serum GGT was indicative of shorter survival in advanced PDAC patients.

Contrarily, systemic inflammation suppresses the synthesis of ALB.^31^ On one hand, the pro‐inflammatory cytokines released by the hepatocytes, such as interleukin‐6 (IL‐6), can negatively regulate the production of ALB and contribute to its decreased serum concentration, independent of patients’ nutrition status. On the other hand, cytokines like tumor necrosis factor (TNF) can increase the permeability of the blood vessel walls, thus promoting the loss of ALB from the circulation.^31^ Subsequent hypoalbuminemia has been demonstrated to be correlated with reduced survival of patients in different types of cancer.^32^, ^33^ With respect to pancreatic cancer, Siddiqui et al^34^ demonstrated that low serum ALB could independently predict poor survival of <6 months in pancreatic cancer patients. Another study also confirmed that in stage IV PDAC patients treated with bevacizumab, those with normal range of ALB had significantly better survival compared with those who had hypoalbuminemia.^23^

For these reasons, GAR is not merely a combination of parameters of liver function test as initially regarded, it acts more as a reflection of internal inflammation status and seems to be useful for estimation of survival in patients with PDAC. In this study, we first analyzed the correlations of GAR and clinicopathologic characteristics and we found that GAR was closely correlated with tumor location, tumor size, vascular invasion, obstructive jaundice, biliary drainage, ALP, ALT, AST, GGT, and ALB. These data indicated that GAR could represent the status of liver function and reflect tumor burden to some extent. In accordance with our hypothesis, univariate analysis revealed that high level of GAR was significantly predictive of poor prognosis for PDAC patients, demonstrated by 7.4 months decrease in OS and 4.8 months decrease in RFS compared with patients who had low level of GAR. The 1, 2, 3‐year OS rates and RFS rates were also markedly lower in patients with high level of GAR compared with those in the low‐level group. After multivariate analysis, the prognostic value of GAR still remained. Subgroup analysis demonstrated that GAR was a significant prognostic factor in patients without abnormalities of obstructive jaundice, GGT, or ALB, and in patients who did not have preoperative biliary drainage. This result indicated that the predictive efficacy of GAR was likely to be limited when patients had preoperative jaundice and impaired liver function. This is a common phenomenon in the current existing prognostic biomarkers for pancreatic cancer. For example, as the most well‐established predictive biomarker, CA19‐9 levels are often elevated in the presence of obstructive jaundice and some benign conditions, which limits its use in clinical practice.^35^ Similarly, a research showed that another inflammation‐based indicator, the systemic immune inflammation index (SIII) could independently predict survival and recurrence in pancreatic cancer patients with normal bilirubin levels, whereas no association between SIII and survival was found in patients with high bilirubin levels.^36^ Taken together, our results demonstrate that high level of GAR is an independent predictor of poor OS and RFS in PDAC patients, but only in the setting of no preoperative obstructive jaundice. From this perspective, GAR should be used with caution in patients with high preoperative bilirubin levels or severely impaired liver function, until more prospective studies support or reject this hypothesis.

Currently, the most reliable prognostic biomarkers, such as the TNM staging system, mainly focus on tumor tissue itself. However, it is widely recognized that not only the intrinsic properties of tumor, but also the host‐related factors, are closely associated with the prognosis of patients after surgery. The integrated index GAR comprehensively reflects the balance of host inflammatory and inflammation status, which may provide more prognostic information from the host perspective. Thus, it is interesting that pretherapeutically available host‐related indicator GAR can synergize with pre‐existing biomarkers, and our results indeed showed that combining GAR and other significant prognostic factors could enhance the prognostic accuracy. In addition, GAR is easily calculated from preoperative parameters of liver function test for each patient, which is money saving and time saving.

Apart from its prognostic value, GAR may also serve several important functions in personalized therapy. First, neoadjuvant therapy is increasingly being employed for borderline resectable pancreatic cancer. Some inflammation‐based biomarkers have been demonstrated to be correlated with patient response to neoadjuvant treatment. For example, Hasegawa et al^37^ reported that neutrophil/lymphocyte ratio (NLR) was significantly higher in pancreatic cancer patients who responded poorly to preoperative chemoradiotherapy compared with those who had a favorable response. This indicates that GAR may also well be a candidate biomarker in evaluating the response to neoadjuvant chemotherapy in PDAC patients. Second, it has been proved in a mouse model of pancreatic cancer that systemic inflammation can diminish the effect of gemcitabine and may thus affect patient survival by altering the response to chemotherapy.^38^ In this regard, GAR can help clinicians identify those who are likely to benefit the most from postoperative chemotherapy. Third, immunotherapy represents a new therapeutic modality that complements conventional chemotherapies without increasing toxicity. As an indicator of systemic inflammation status, GAR may be useful in selecting appropriate patients for immunotherapy. Many studies have demonstrated the value of inflammatory biomarkers in predicting patient response to immunotherapy in different types of tumor.^39^ It is therefore interesting to investigate whether GAR can become a potential predictive biomarker for pancreatic cancer immunotherapy clinical trials in the future.

However, three limitations need to be taken into account in this study. First, this is a retrospective analysis and all the clinical data were collected from a single institution in China. Whether the cut‐off value of GAR proposed by our study is suitable for other institutions and patient populations remain to be validated. A larger‐scale prospective study with multicenter involved is needed to further verify our findings. Second, our study only includes patients underwent radical surgery, without considering those with unresectable PDAC or the impact of different postoperative treatments. Third, GAR has the potential to become a prognostic indicator, but only in the setting of those without preoperative biliary obstruction. GAR may lose its predictive value in patients who have obstructive jaundice and severely impaired liver function.

In conclusion, our study demonstrates that as a novel and easily accessible ratio index, preoperative GAR can be used as a prognostic factor for predicting the prognosis of patients with PDAC after radical resection. Combination of TNM stage, GAR, preoperative serum CA19‐9, and tumor differentiation can enhance the prognostic accuracy for survival prediction. Further independent prospective clinical trials should be evaluated to confirm these results.

## CONFLICT OF INTEREST

The authors declare that they have no conflict of interests.
